# The effect of non-opioid multimodal analgesics and dexamethasone monotherapy on acute incisional pain behaviors in rats

**DOI:** 10.3389/fpain.2025.1569246

**Published:** 2025-08-13

**Authors:** Ratan K. Banik, Malcolm E. Johns, Twan Sia, Donald A. Simone

**Affiliations:** ^1^ Department of Anesthesiology, School of Medicine, University of Minnesota, Minneapolis, MN, United States; ^2^ Stanford University School of Medicine, Stanford, CA, United States; ^3^ Department of Diagnostic and Biological Sciences, School of Dentistry, University of Minnesota, Minneapolis, MN, United States

**Keywords:** nonopioid, multimodal, rat, postoperative pain, incision

## Abstract

The use of non-opioid multimodal analgesics (NMA) may enhance pain relief and decrease opioid dependence in managing acute incisional pain, although this remains debated. A clinical trial found NMA ineffective compared to placebo, prompting us to investigate its impact on pain-like behaviors in animal models. In our study, 12 rats underwent plantar incision surgery and were divided into two groups: NMA and vehicle. NMA comprised acetaminophen, celecoxib, gabapentin, and dextromethorphan, with dosages based on human equivalents. We measured paw withdrawal latency (PWL), paw withdrawal threshold (PWT), and spontaneous foot lifting (SFL) behaviors. Before injection, there were no significant differences between the groups in PWL, PWT, or SFL. After treatment, PWL increased in NMA-injected rats (9.8 ± 2.2 s) compared to vehicle (5.9 ± 2.7 s; *p* = 0.02). SFL frequency decreased in NMA-injected rats (8.0 ± 5.0 count/20-min) vs. vehicle (30.7 ± 18.0 count/20-min; *p* = 0.013). However, PWT and SFL duration showed no significant changes. This research represents the first exploration of NMA's effects on incisional pain, suggesting it may effectively manage acute postsurgical pain with inflammatory and neuropathic components. Further clinical validation is needed, but our results indicate NMA could be a viable opioid alternative.

## Introduction

Postoperative, incisional pain is a unique but common form of acute pain. Approximately 310 million major surgical procedures are performed every year worldwide (1). Currently, opioids are the mainstay for perioperative pain management, however, they have significant side effects (2). In 2018, Opioids were involved in approximately 70% (46,802) of drug overdose deaths during 2018 (3). The misuse of and addiction to opioids—including prescription opioid is a serious national crisis (4). The Centers for Disease Control and Prevention estimates that the total “economic burden” of prescription opioid misuse alone in the United States is $78.5 billion a year (5), including the costs of healthcare, lost productivity, addiction treatment, and criminal justice involvement. Unfortunately, attempts for discovery of a potent non-opioid analgesic for acute postoperative pain has not been successful despite billions of dollars have been spent in research. Moreover, drug development is becoming increasingly time-consuming (an average of 9–12 years for new drugs), There is, therefore, an urgent critical need for investigation on drug repurposing, aiming to discover new uses of existing non-opioid drugs for postsurgical pain.

Previous work has demonstrated the clinical utility of acetaminophen (6, 7), gabapentin [25], N-methyl-d-aspartate (NMDA) blockers (8), and celecoxib (9) in controlling postoperative pain. A Cochrane review (51 studies) showed about half of participants treated with acetaminophen achieved at least 50% pain relief over 4–6 h, compared with about 20% treated with placebo (6). In a meta-analysis (27 randomized clinical trials), the VAS pain score and opioid consumption was significantly reduced with gabapentin vs. placebo (10). A systematic review (17 studies) on NMDA antagonists showed a reduction in total opioid consumption and increase in time to first analgesia across all studies (8). Selective cyclooxygenase (COX)-2 inhibitor celecoxib demonstrated efficacy in acute postoperative pain in a Cochrane review (10 studies) (9).

Thus, we hypothesize that the combined use of these non-opioid analgesic drugs may provide analgesic effects in the setting of acute incisional pain and thus may be opioid sparing. However, previous clinical studies have shown mixed results. In patients undergoing cardiac surgery, a multimodal regimen provided significantly better analgesia compared to a traditional opioid-based approach (11). Similarly, opioid-sparing multimodal pain management protocols achieved acceptable pain control following transsphenoidal surgery (12). In contrast, a perioperative multimodal regimen did not reduce opioid consumption within 48 h after cesarean section (13). Furthermore, the use of a multimodal analgesic approach failed to improve Day 3 quality of recovery, pain scores, or 48-hour opioid use in other settings (14).

In this study, we compared effects of the combined use non-opioid multimodal analgesic drugs (NMA) comprised of acetaminophen, celecoxib, an NMDA blocker (dextromethorphan), and gabapentin vs. vehicle on incisional pain behaviors in a rat model.

Secondarily, dexamethasone is frequently administered to prevent postoperative nausea and vomiting. In a meta-analysis of 24 clinical trials with 2,751 subjects, a single dose of dexamethasone (>0.1 mg/kg) was shown to reduced postoperative pain and opioid consumption after surgery when administered with other analgesics [7]. Thus, we also compared the effects of dexamethasone alone vs. vehicle on incisional pain behaviors.

## Methods

### Animals

All studies were approved by the University of Minnesota Institutional Animal Care and Use Committee (1905-37106A, approval date, July 19, 2019). Adult male Sprague-Dawley rats (250–300 g) were purchased from Harlan (Somerville, NJ). There were no exclusion criteria for experimentation or analysis. Rats were housed in pairs in polymethyl methacrylate cages (43 × 21.5 × 25.5 cm) and kept on 12-h light/dark cycle. Food and water were available *ad libitum*. None of animals were excluded from this study.

### Plantar incision

To generate the incisional pain model, rats were anesthetized with isoflurane before surgical incision. Each animal was placed in a plexiglass induction chamber containing 5% isoflurane in room air. Upon righting reflex loss, 2%–3% isoflurane in room air was delivered through a nose cone. A 20-mm longitudinal incision was made through the skin and fascia of the plantar hind paw. The plantaris muscle was elevated, stressed, and incised longitudinally. The origin and insertion of the muscle remained intact. The skin was closed using 2 mattress 5-0 silk suture.

### Administration of nonopioid multimodal analgesia, dexamethasone, or vehicle

Six rats each were given a single intraperitoneal injection of NMA, dexamethasone, or respective vehicle (24 rats total). NMA or vehicle was administered following surgery, and dexamethasone or vehicle was administered 30 min before surgery. Rats were randomized into treatment groups without considering any other variables. NMA was comprised of acetaminophen (90 mg/kg in 30% polyethylene glycol/saline), celecoxib (15 mg/kg in 60% ethanol/40% polyethylene glycol), gabapentin (50 mg/kg in saline), and dextromethorphan (15 mg/kg in saline). Dexamethasone concentration was 1 mg/kg in saline. Doses were determined by converting doses administered in humans using allometric scaling [18]. Thus, drug dose is based on normalization of dose to body surface area and unique characteristics of anatomical, physiological, and biochemical process among species [18]. Moreover, the experimental protocol for drug administration was designed based on the peak pharmacological effects of NMA and the known pharmacokinetics of the administered drugs. Acetaminophen reaches peak serum concentration within 1–2 h after oral administration (15), while gabapentin peaks at approximately 3–4 h (16). Dextromethorphan and celecoxib (Celebrex) typically exhibit peak effects within 2–3 h (17, 18). In contrast, dexamethasone, a long-acting corticosteroid, has a biological half-life of 36–54 h (19).

### Pain behavior measurement

**Figure 1:**
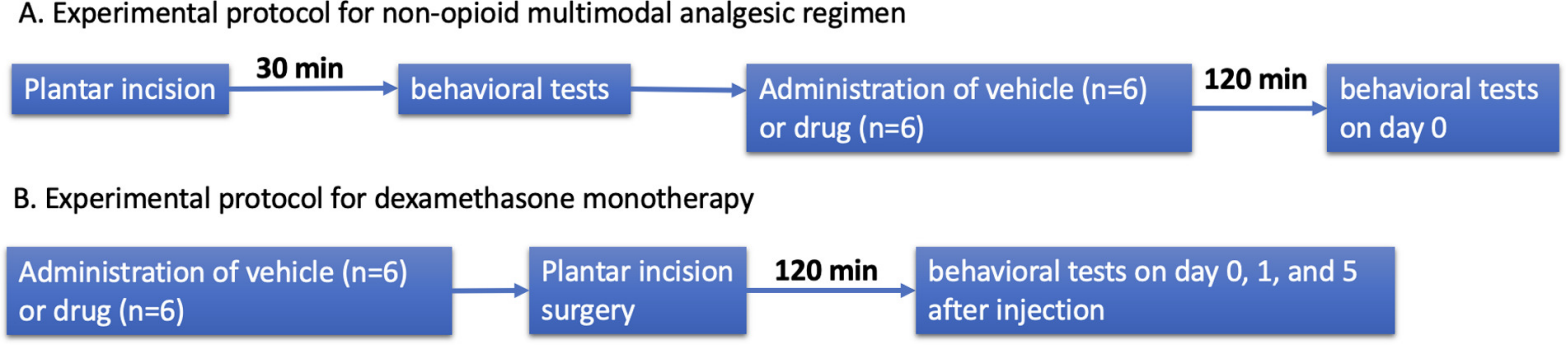
Experimental protocol. **(A)** Experimental protocol for non-opioid multimodal analgesic regimen. **(B)** Experimental protocol for dexamethasone monotherapy.

Researchers were blinded to what treatments were administered while measuring pain behaviors. Spontaneous pain behavior was measured by assessing spontaneous foot lifting (SFL) frequency and duration. Heat hyperalgesia was assessed by measuring paw withdrawal latency (PWL) using the Hargreaves test. Mechanical allodynia was assessed by measuring paw withdrawal threshold (PWT) using the electronic von Frey test. Detailed methods are available in the Supplemental methods [1,2,11]. Experimental protocol is described in Figure 1.

### Statistical analysis

Pre- and post-injection PWL, PWT, and SFL frequency and duration were compared in NMA- and vehicle-injected rats with unpaired *t*-tests. PWL and SFL frequency and duration on day 0, 1, and 5 after injection were compared in dexamethasone- vs. vehicle-injected rats with two-way analyses of variance with repeated measures. Analyses were performed using Prism 10 (GraphPad Software, San Diego, CA). *P*-value of <0.05 were considered significant. Data are presented as means ± standard deviation.

## Results

**Figure 2:**
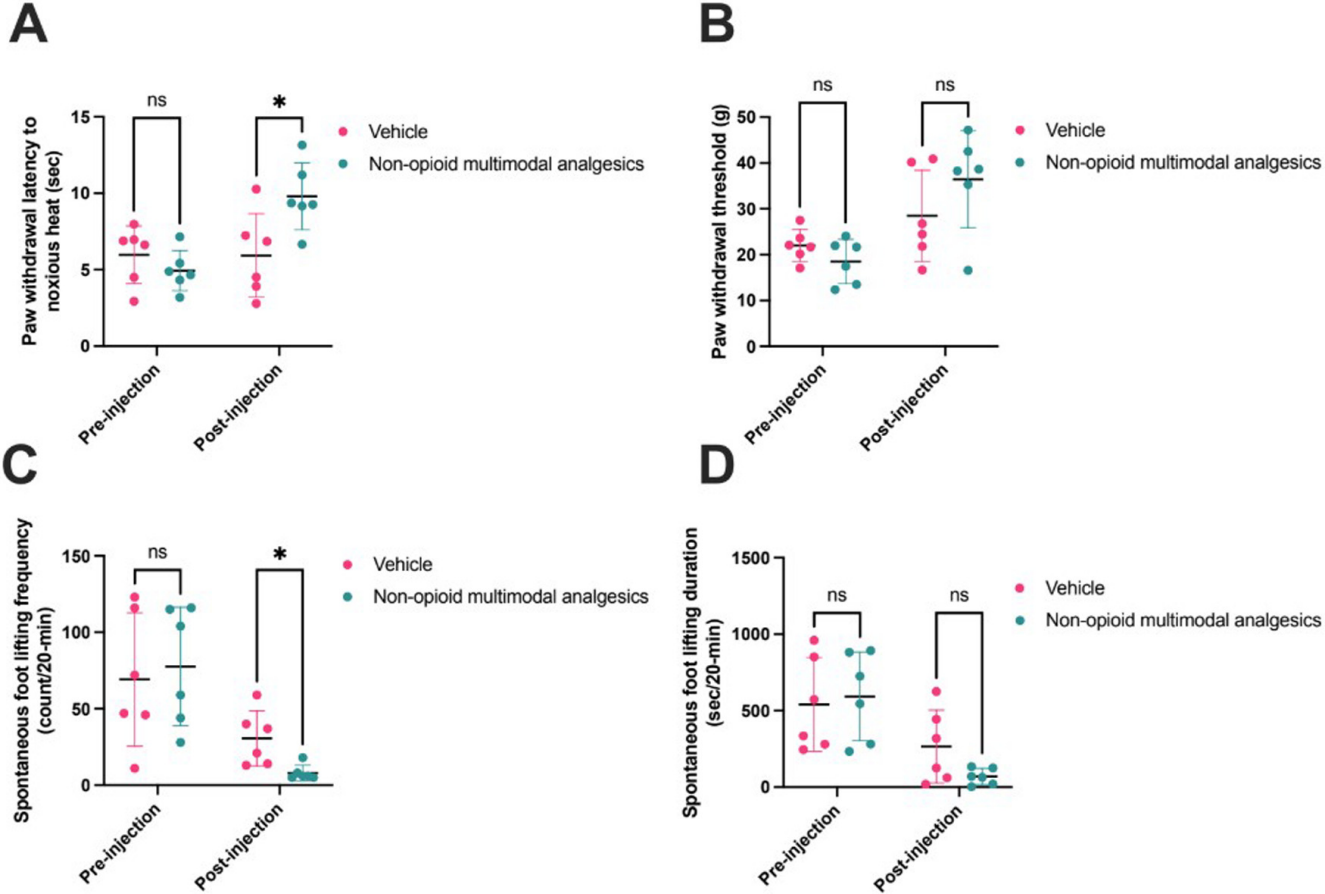
Non-opioid multimodal analgesia (*n* = 6) versus vehicle (*n* = 6) pre- and post-injection in rat incisional pain models. Pain behaviors were assessed by **(A)** Hargreaves test (pre-injection *p* = 0.29; post-injection *p* = 0.02), **(B)** von Frey test (pre-injection *p* = 0.18; post-injection *p* = 0.21), and **(C)** spontaneous foot lifting frequency (pre-injection *p* = 0.73; post-injection *p* = 0.01) and **(D)** duration (pre-injection *p* = 0.77; post-injection *p* = 0.08). Comparisons were made using unpaired *t*-tests. Horizontal lines represent means, and error bars represent standard deviation. * denotes *p* < 0.05.

Before injection of NMA (*n* = 6) or vehicle (*n* = 6), there was no difference in paw withdrawal latency (PWL; NMA 4.9 ± 1.3 s, vehicle 6.0 ± 0.8 s; *p* = 0.29), paw withdrawal threshold (PWT; NMA 18.5 ± 4.8 g, vehicle 22.0 ± 3.5 g; *p* = 0.18), or SFL frequency (NMA 77.7 ± 38.7 count/20-min, vehicle 69.2 ± 43.6 count/20-min; *p* = 0.73) or duration (NMA 593.7 ± 289.4 s/20-min, vehicle 541.2 ± 306.7 s/20-min; *p* = 0.77). After injection, PWL was increased following NMA (9.8 ± 2.2 s). An increase in PWL was not observed in vehicle-injected rats (5.9 ± 2.7 s; *p* = 0.02). NMA decreased SFL frequency (8.0 ± 5.0 count/20-min) as compared to vehicle (30.7 ± 18.0 count/20-min; *p* = 0.013). In contrast, PWT (NMA 18.5 ± 4.8 g, vehicle 28.5 ± 9.9; *p* = 0.21) and SFL duration (NMA 69.7 ± 53.2 s/20-min, vehicle 265.8 ± 239.0 s/20-min; *p* = 0.078) were not changed after NMA (Figure 2).

**Figure 3:**
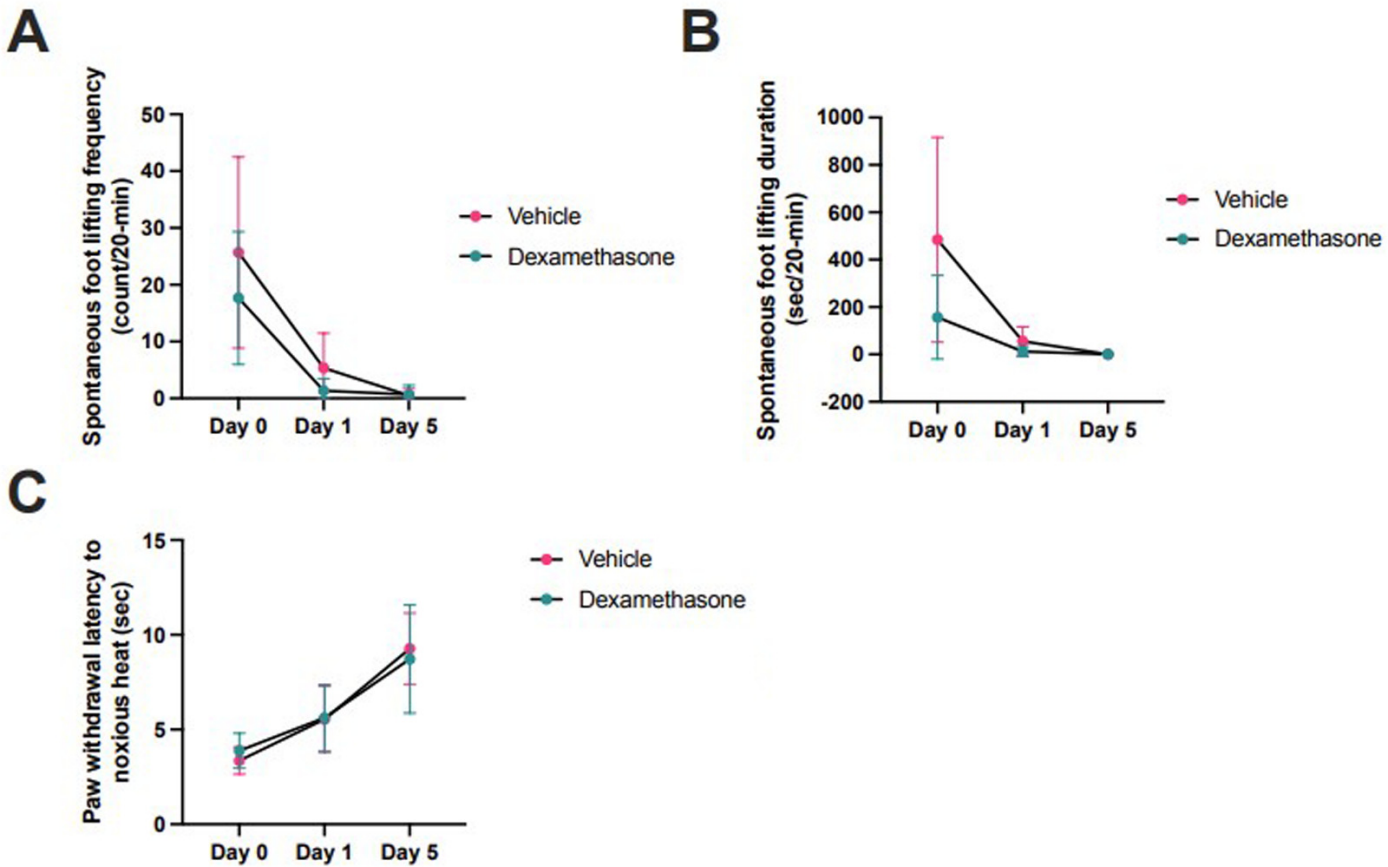
Dexamethasone (*n* = 6) versus vehicle (*n* = 6) at day 0, 1, and 5 injection in rat incisional pain models. Pain behaviors were assessed by **(A)** spontaneous foot lifting frequency [2-way ANOVA F(1,10) = 2.2, *p* = 0.16] and **(B)** duration [2-way ANOVA F(1, 10) = 0.0007, *p* = 0.98], and **(C)** Hargreaves test [2-way ANOVA F(1, 10) = 3.4, *p* = 0.10]. Comparisons were made using 2-way ANOVAs (analysis of variance). Horizontal lines represent means, and error bars represent standard deviation. The horizontal axis is not linearly scaled with time.

In another group of animals, effects of dexamethasone (*n* = 6) or vehicle (*n* = 6) were assessed on day 0, 1, and 5 after injection. Over the measurement period, there were no significant differences in SFL count [2-way ANOVA F(1,10) = 2.2, *p* = 0.16], SFL duration [2-way ANOVA F(1, 10) = 0.0007, *p* = 0.98], or PWL [2-way ANOVA F(1, 10) = 3.4, *p* = 0.10] between dexamethasone- and vehicle-treated rats. However, SFL duration (dexamethasone 139 ± 69 count/20-min, vehicle 736 ± 100 count/20-min; *p* = 0.08) and frequency (dexamethasone 12 ± 21 s/20-min, vehicle 45 ± 29 s/20-min; *p* = 0.37) tended to decrease following dexamethasone, but this was not statistically significant (Figure 3).

## Discussion

NMA for postoperative pain has been suggested by the American Society of Anesthesiologists Taskforce on Acute Pain Management (20), but NMA remains underutilized. In a study of 799,449 patients who underwent a procedure at 315 hospitals in the USA, 97% received an opioid, whereas 66% received acetaminophen (21). In this background, this preclinical study suggests NMA can effectively reduce pain-like behaviors after plantar incision in an animal model (Figure 2). This baseline data obtained prior to the drug administration demonstrates that the observed differences following drug or saline administration are not attributable to pre-existing disparities between groups. Rather, the changes emerge only after treatment is initiated, supporting the conclusion that the drug effects are responsible for the group differences.

Postoperative incisional pain has a distinct pathophysiology with components of both inflammatory and neuropathic pain. Following tissue incision, inflammatory mediators are released locally and systemically, contributing to nociceptive sensitization (22, 23). Small nerves injured during surgery can discharge spontaneously, which can evolve into chronic neuropathic pain. Thus, it is reasonable to suggest NMA comprised of cyclooxygenase inhibitors (acetaminophen, celecoxib), anti-neuropathic agents (gabapentin), and drugs counteracting central sensitization (NMDA blocker) to treat acute postsurgical pain.

Our results contrast with a clinical trial on spine surgery patients by Maheshwari et al., showing that NMA was not superior to placebo (14). There are several possible explanations. First, we tested animals within 2 h of drug administration, when there is a peak effect of these drugs. Maheshwari et al. measured their endpoints over the 48-hour period post-surgery (14). Given that all NMA drugs have half-lives of less than 8 h (15–19), it is likely that they were no longer pharmacologically active at the time of measurement. Secondly, the NMA composition is also different between studies: celecoxib was not included by Maheswari et al. (14)which has been demonstrated to be effective in acute pain in a Cochrane review (9).

Another advantage of NMA is their potential to prevent persistent postsurgical pain (24). Several components of NMA such as gabapentin, pregabalin, and NMDA blockers have been shown to be effective in suppressing central sensitization and have been beneficial for reducing persistent postsurgical pain in several clinical trials (8, 24, 25). Here, we were unable to study the effects of NMA on persistent postsurgical pain, as spontaneous pain behaviors in our animal model are short-lasting (26). However, the effects of several NMA components, such as gabapentinoids has been well-documented in the literature. In several well-designed clinical trials, perioperative gabapentinoids (gabapentin and pregalbalin) have been shown to prevent persistent postsurgical pain or improve quality of life after total knee arthroplasty (27), spine surgery (25, 28), lumbar discectomy (29), and hysterectomy (30) at 3 and 6 months after surgery.

Pain behaviors were not different in dexamethasone- and vehicle-injected rats, however, spontaneous pain behaviors tended to be decreased 2 h after surgery. Dexamethasone as an adjunctive therapy reduced postoperative pain and opioid consumption after surgery compared in placebo (31). Thus, dexamethasone may be a useful synergistic addition to NMA, though further studies are needed to confirm.

## Limitation

The study has inherent limitations of animal studies and challenges in translating preclinical findings into effective human therapies (32). One of the most significant issues is the biological and physiological differences between animal models and humans, which can limit the predictive validity of our studies. For instance, rodents often respond differently to pain stimuli, inflammation, or pharmacologic interventions due to species-specific differences in receptor expression, metabolism, and immune responses. Furthermore, we used young, healthy animals housed in controlled environments, which may not reflect the heterogeneity and comorbidities present in human patients (33). Additionally, behavioral outcomes used in animal studies—such as withdrawal thresholds or reflexive responses—may not adequately capture the complex, subjective experience of pain in humans (34). Other limitations of our study include the fact that we did not investigate the potential adverse effects of NMA. Though the detrimental effects of opioids are relatively well-documented, non-opioid analgesics are not completely harmless. Thus, the potential safety profile of NMA needs to be investigated as well.

## Conclusion

In conclusion, our findings demonstrate that NMA administration significantly reduces heat hyperalgesia and spontaneous pain behaviors following incision, without affecting mechanical allodynia. Importantly, baseline pain behaviors did not differ between NMA- and vehicle-treated groups, indicating that the observed post-treatment effects are not due to pre-existing differences but rather are attributable to the pharmacological action of NMA. To our knowledge, this is the first basic science study to investigate the effects of NMA on incisional pain behaviors, providing novel evidence for its potential as a targeted analgesic strategy.

## Data Availability

The original contributions presented in the study are included in the article/Supplementary Material, further inquiries can be directed to the corresponding author.
